# Supplementary material for: Prevalence of Childhood Asthma and Allergies and Their Associations with Perinatal Exposure to Home Environmental Factors: A Cross-Sectional Study in Tianjin, China
Source: Int J Environ Res Public Health. 2021 Apr 14;18(8):4131. doi: 10.3390/ijerph18084131 (PMC8070727; doi:10.3390/ijerph18084131)
Supplement: Supplementary file 1 [file ijerph-18-04131-s001.zip › Supplementary/Supplementary material table 1.docx]

Supplementary material

Prevalence of Childhood Asthma and Allergies and Their Associations with Perinatal Exposure to Home Environmental factors: A Cross Sectional Study in Tianjin, China

Agnes S. Ellie, Yuexia Sun *, Jing Hou, Pan Wang, Qingnan Zhang and Jan Sundell

**Table S1: Associations between perinatal factors and children's allergic diseases using binary logistic regression without adjustments**

| **Factor** | **DDA** | **Dry cough** | **Current wheeze** | **DDR** | **Current rhinitis** | **DDE** | **Current eczema** |
| --- | --- | --- | --- | --- | --- | --- | --- |
|  | **OR(95%CI)** | **OR(95%CI)** | **OR(95%CI)** | **OR(95%CI)** | **OR(95%CI)** | **OR(95%CI)** | **OR(95%CI)** |
| **Dampness (mold)** | | | |  |  |  |  |
| Visible mold or damp stain | 2.08(1.46–2.97)^a^ | 1.69(1.33–2.14)^a^ | 2.36(1.68–3.31)^a^ | 1.73(1.31–2.27)^a^ | 1.92(1.58–2.33)^a^ | 1.46(1.21–1.76)^a^ | 1.77(1.40–2.23)^a^ |
| Carpet peeling up or discolored | 1.47(0.96–2.26) | 1.67(1.27–2.18)^a^ | 1.63(1.08–2.45)^a^ | 1.56(1.14–2.14)^a^ | 1.64(1.32–2.05)^a^ | 1.27(1.02–1.5) | 1.54(1.18–2.02)^a^ |
| Flooding damage | 2.68(1.70–4.21)^a^ | 2.02(1.45–2.80)^a^ | 2.47(1.57–3.88)^a^ | 2.59(1.81–3.68)^a^ | 1.92(1.45–2.55)^a^ | 1.34(1.01–1.78)^a^ | 1.25(0.87–1.81) |
| Condensation on windows | 1.43(1.04–1.96)^a^ | 1.26(1.02–1.55)^a^ | 1.80(1.33–2.44)^a^ | 1.50(1.19–1.89)^a^ | 1.45(1.24–1.70)^a^ | 1.00(1.38–1.86)^a^ | 1.70(1.40–2.07)^a^ |
| Suspected moisture problem | 2.12(1.52–2.97)^a^ | 1.51(1.20–1.91)^a^ | 2.09(1.49–2.92)^a^ | 1.69(1.30–2.19)^a^ | 1.63(1.36–1.96)^a^ | 1.61(1.35–1.93)^a^ | 1.88(1.51–2.34)^a^ |
| **Humidity and odor** | | | |  |  |  |  |
| Stuffy smell | 1.40(1.01–1.94)^a^ | 1.61(1.31–1.97)^a^ | 1.67(1.22–2.28)^a^ | 1.70(1.35–2.15)^a^ | 1.87(1.60–2.19)^a^ | 1.85(1.59–2.16)^a^ | 2.06(1.70–2.50)^a^ |
| Unpleasant smell | 1.61(1.10–2.37)^a^ | 1.70(1.33–2.17)^a^ | 2.19(1.53–3.12)^a^ | 1.69(1.27–2.25)^a^ | 1.78(1.46–2.18)^a^ | 1.60(1.32–1.95)^a^ | 1.51(1.17–1.93)^a^ |
| Pungent smell | 2.02(1.18–3.45)^a^ | 1.53(1.05–2.22)^a^ | 2.73(1.70–4.38)^a^ | 1.89(1.26–2.84)^a^ | 1.71(1.26–2.32)^a^ | 1.54(1.40–2.07)^a^ | 1.95(1.38–2.76)^a^ |
| Mildew smell | 2.00(1.27–3.16)^a^ | 1.77(1.30–2.41)^a^ | 2.11(1.35–3.31)^a^ | 1.89(1.33–3.69)^a^ | 2.31(1.79–2.99)^a^ | 1.80(1.40–2.32)^a^ | 2.44(1.83–3.25)^a^ |
| Perceived dry air | 1.37(1.00–1.87)^a^ | 1.60(1.31–1.95)^a^ | 1.62(1.19–2.21)^a^ | 1.52(1.21–1.92)^a^ | 1.81(1.56–2.11)^a^ | 1.73(1.50–2.01)^a^ | 1.70(1.40–2.06)^a^ |
| Perceived Humid air | 1.52(1.06–2.18)^a^ | 1.40(1.10–1.77)^a^ | 1.36(0.93–1.98) | 1.41(1.07–1.86)^a^ | 1.53(1.27–1.84)^a^ | 1.69(1.42–2.03)^a^ | 1.81(1.45–2.27)^a^ |
| **Parent smoking** | | | |  |  |  |  |
| FSP | 1.16(0.91–1.47) | 1.03(0.89–1.19) | 1.28(1.01–1.61)^a^ | 0.99(0.83–1.18) | 0.99(0.88–1.10) | 1.06(0.96–1.17) | 1.06(0.92–1.22) |
| FSF | 1.08(0.85–1.37) | 0.99(0.86–1.15) | 1.12(0.89–1.41) | 1.02(0.86–1.21) | 0.98(0.88–1.09) | 1.02(0.92–1.12) | 1.01(0.88–1.16) |
| MSP | 2.62(1.24–5.54)^a^ | 1.29(0.71–2.35) | 3.63(1.89–7.00)^a^ | 1.33(0.66–2.68) | 0.89(0.53–1.47) | 1.44(0.90–2.31) | 1.30(0.73–2.34) |
| MSF | 3.53(1.84–6.79)^a^ | 1.38(0.78–2.42) | 3.07(1.61–5.88)^a^ | 2.36(1.33–4.18)^a^ | 0.80(0.49–1.33) | 1.21(0.77–1.90) | 1.06(0.58–1.93) |
| **Contact with animals** | | | |  |  |  |  |
| Cat | 1.85(0.96–3.56) | 1.45(0.92–2.25) | 1.80(0.93–3.47) | 1.28(0.74–2.20) | 1.02(0.70–1.50 | 1.06(0.74–1.52) | 1.45(0.94–2.24) |
| Dog | 0.51(0.31–0.84)^a^ | 0.99(0.78–1.24) | 0.86(0.58–1.27) | 0.91(0.69–1.21) | 0.84(0.70–1.00) | 0.92(0.78–1.09) | 1.18(0.95–1.46) |
| **Renovation** | | | |  |  |  |  |
| FCR | 1.27(0.73–2.21) | 1.61(1.18–2.19)^a^ | 2.13(1.39–3.27)^a^ | 1.74(1.23–2.47)^a^ | 1.32(1.02–1.71) | 1.10(0.86–1.42) | 0.65(1.22–2.22)^a^ |
| FPR | 1.28(0.79–2.08) | 1.49(1.12–1.98)^a^ | 1.66(1.07–2.59) | 1.61(1.15–2.24)^a^ | 1.04(0.90–1.20) | 1.32(1.07–1.64) | 1.73(1.32–2.28)^a^ |
| PCR | 1.48(0.99–2.22) | 1.35(1.05–1.74)^a^ | 1.62(1.12–2.36)^a^ | 1.43(1.07–1.91)^a^ | 1.35(1.11–1.65)^a^ | 0.95(0.78–1.15) | 1.20(0.93–1.54) |
| PPR | 1.06(0.76–1.47) | 1.37(1.09–1.72)^a^ | 1.28(0.88–1.86) | 1.31(1.00–1.72)^a^ | 0.99(0.89–1.11) | 1.00(0.90–1.11) | 1.24(0.99–1.56) |

DDA = Doctor-diagnosed asthma, DDR = doctor-diagnosed, Rhinitis, DDE = doctor-diagnosed eczema, FSF= father smoking during the child's first year of life, FSP= father smoking during pregnancy, MSP= mother smoking during pregnancy, MSF= mother smoking during the child's first year of life, FCR= floor in child's room, FPR= floor in parents' room, PCR= paint/wall in child's room, PPR= paint/wall in parents' room. Odd ratios of the variables were calculated in binary logistic regression. (^a^ )represents *p*-value <0.05.
